# Comment on Öztürk et al. Association Between Fresh Gas Flow Rate and Optic Nerve Sheath Diameter During Laparoscopic Cholecystectomy: A Prospective Observational Study. *J. Clin. Med*. 2026, *15*, 7626

**DOI:** 10.3390/jcm15218327

**Published:** 2026-10-26

**Authors:** Ali Akdogan, Sedat Saylan, Ersagun Tugcugil, Ahmet Besir

**Affiliations:** Department of Anesthesiology and Reanimation, Faculty of Medicine, Karadeniz Technical University, Trabzon 61000, Türkiye

We read with interest the prospective observational study by Öztürk et al. [1], who compared perioperative optic nerve sheath diameter (ONSD) trajectories at fresh gas flow rates of 1 and 3 L/min during desflurane anesthesia for laparoscopic cholecystectomy. We commend the blinded ONSD assessments, the longitudinal analysis, and the authors’ caution that a nonsignificant result does not establish equivalence. We would welcome clarification of remifentanil exposure to help interpret the reported anesthetic concentration differences and contextualize the ONSD findings.

The age-adjusted minimum alveolar concentration (MAC) displayed by the anesthesia workstation was lower in the low-flow group at T2 (5 min after pneumoperitoneum; 0.66 ± 0.06 versus 0.73 ± 0.09) and T3 (20 min after pneumoperitoneum; 0.68 ± 0.06 versus 0.74 ± 0.10), with a significant group × time interaction. No significant group effect or group × time interaction was detected for the bispectral index (BIS) [1]. The authors suggest that the MAC difference may reflect flow-dependent anesthetic delivery and equilibration. They also note that the regimen included remifentanil (0.05–0.2 µg/kg/min) titrated, with desflurane, to BIS and physiological targets and that the MAC difference should be interpreted in the context of the overall regimen [1]. However, neither infusion rates nor cumulative remifentanil doses are reported by group.

Remifentanil reduces the desflurane concentration required to blunt sympathetic responses to incision, as demonstrated by Albertin et al. using the minimum alveolar concentration blocking adrenergic responses (MAC-BAR) in the presence of nitrous oxide [2]. This endpoint differs from BIS-guided hypnosis and from the displayed age-adjusted MAC; its effect size cannot be transferred directly to the present oxygen–air regimen. In a different experimental setting, Jung et al. found no significant between-group BIS difference after administering remifentanil or saline to women with propofol-induced unconsciousness in the absence of noxious stimulation [3]. Although that setting differs from desflurane maintenance, it illustrates why similar BIS values alone cannot establish comparable opioid exposure.

Differences in remifentanil titration could therefore contribute to the achieved desflurane concentrations, alongside flow-dependent delivery and equilibration. The available data establish neither the presence nor the direction of such differences. We emphasize that any implication for the primary outcome is indirect: we do not suggest that differences in anesthetic or opioid exposure necessarily influenced ONSD or intracranial pressure, and an opioid-related explanation for the MAC findings would not by itself demonstrate such an effect. Rather, because the conclusions are framed as applying “under the studied conditions” [1], drug exposure forms part of the context that readers need to evaluate. Clarifying it would help readers assess whether the two flow strategies were observed under comparable anesthetic conditions.

We therefore suggest reporting remifentanil infusion rates by group at the intraoperative ONSD assessments, particularly T2 and T3. Weight-normalized cumulative doses over the preceding measurement intervals would be a useful addition only if these records are readily available; their absence would not by itself compromise the interpretation of the original findings. Inspired and end-tidal desflurane concentrations at the same assessments would also help characterize delivery and achieved volatile exposure. We regard these data primarily as descriptive. Because remifentanil was titrated to intraoperative responses and may itself have been influenced by flow-related changes in desflurane delivery, any exposure-adjusted analysis would require careful consideration of this temporal relationship and should be interpreted as exploratory.

We thank the authors for contributing prospective data on a clinically relevant question. This clarification would strengthen the interpretation of the MAC and BIS findings and the anesthetic context of the ONSD comparison while preserving the study’s appropriately cautious conclusions.

## Data Availability

No new data were created or analyzed in this study. Data sharing is not applicable to this article.
